# A New Surgical Technique and Clinical Outcomes of Operated Proximal Metadiaphyseal Humeral Fractures and Nonunions With the Use of Reversed Anatomic Distal Femoral Locking Plate

**DOI:** 10.7759/cureus.18309

**Published:** 2021-09-27

**Authors:** Byron Chalidis, Dimitrios Kitridis, Panagiotis Givissis

**Affiliations:** 1 School of Medicine-1st Orthopaedic Department, Aristotle University of Thessaloniki, Thessaloniki, GRC

**Keywords:** deltopectoral approach, locking plate, nonunion, metadiaphyseal humeral fractures, proximal humerus fracture

## Abstract

Introduction

Proximal humeral fractures complicated with metaphyseal and diaphyseal extension are usually treated operatively with 3.5 mm long anatomic proximal humerus plates. However, frequently these comminuted and segmental fracture types may be associated with delayed union, nonunion, and/or plate failure. We present a technique for addressing this fracture pattern by using an anatomic contralateral 4.5 mm distal femoral plate in a reversed fashion.

Methods

Eleven patients (eight women and three men) with a mean age of 70 years (range, 52 to 84 years) were operated on with the described technique. The dominant hand was involved in seven out of 11 patients. There were seven acute metadiaphyseal fractures and four nonunions. In one patient, humeral shaft nonunion was associated with segmental metadiaphyseal defect and a free fibular graft was applied.

Results

All fractures healed and patients regained almost normal function of the affected shoulder and upper limb. Shoulder abduction and forward elevation ranged from 80 to 110 degrees (mean, 97 degrees) and 90 to 120 degrees (mean, 102 degrees), respectively. The disabilities of the arm, shoulder and hand (DASH) score varied from 6 to 11 points (median 8). No major trauma or systemic complications were recorded.

Conclusion

The morphology, strength, and characteristics of the plate could effectively conform to the anatomy of the proximal humerus and offer adequate stability for fracture union. The described technique is more useful in case of osteoporosis and/or presence of previous failed internal fixation that further compromise the vascularization and the mechanical properties of the bone.

## Introduction

Proximal humerus fractures with diaphyseal extension are a rare and inherently unstable fracture pattern. They mainly occur in osteoporotic bone exhibiting a bending wedge or a long spiral type with or without a large butterfly fragment [1]. A range of treatment options have been described for these fractures, ranging from nonoperative management to internal fixation techniques, including intramedullary nailing and plate fixation [1-7]. However, these fractures are too high for humeral bracing and adequate stability is difficult to be maintained. Furthermore, and due to fracture complexity and comminution, early rehabilitation is delayed, fracture healing is compromised and considerable morbidity may be encountered [4]. Therefore, the fractures involving the metadiaphyseal area of the proximal humerus are preferably treated surgically [2,3].

Intramedullary nailing can be performed without opening the fracture site to avoid damage to the endosteal and periosteal circulation [8]. However, simultaneous reduction of both proximal and distal fracture fragments is difficult especially in comminuted and segmental fracture types involving the head tuberosities [5]. Therefore, closed humeral nailing may predispose to malreduction and further fracture displacement. Moreover, radial nerve palsy is considered also a relevant contraindication of the technique due to suggested nerve exploration whereas rotator cuff integrity might be compromised during antegrade nail insertion [5].

Pre-contoured anatomical proximal locking compression plates such as PHILOS (Synthes, Oberdorf, Switzerland) provide angular as well as axial stability and the option of multiple screw insertion in a convergent/ divergent fashion [9,10]. In the case of metadiaphyseal segmental fractures, the plate should extend to the humeral shaft and have adequate length to accomplish at least six cortical fixation points distal to the fracture zone [11]. It can also be twisted in a helical form to aid anterior distal fixation and prevent injury of the radial nerve [12].

On the other hand, implant failure of this construct isn’t rare, and more rigid fixation with 4.5 mm plates and screws is required particularly in osteoporotic bone fractures, nonunions, and previously failed osteosynthesis [13,14]. These plates can also be twisted in a helical form to cover the entire shaft, from the anterolateral to the anteromedial surface of the humerus distally. However, they cannot fit anatomically to the proximal humerus and don’t have the option of multiple screws insertion in the humeral head area. Moreover, the preparation of a helical plate involves some technical difficulties and currently, helical plates for the humerus are not commercially available [7].

To address the above issues, we present a new surgical technique for fixation of the proximal metadiaphyseal humeral fractures and nonunions. A contralateral 4.5 mm distal anatomic locking femoral plate that is used in a reverse fashion can nicely fit the anatomy of the proximal humerus and provide strong fixation for achieving fracture healing.

## Materials and methods

Eleven patients suffering from proximal metadiaphyseal humeral fractures and nonunions were operated on with the described technique between March 2018 to December 2020. Institutional ethics committee approval was obtained for the study (Approval number: 0040/405 Approval date: 28.03.2018). All participants were informed about the study and an informed consent form was obtained.

Operative technique

Under general anesthesia, a beach chair position with the operating table semi-reclined to approximately 45 to 60 degrees was applied. The patient was positioned with the involved shoulder at the edge of the table and the arm supported in approximately 40 to 60 degrees of abduction with a Mayo stand. C-arm fluoroscopy was routinely placed on the same side of the affected extremity and behind the patient’s head. The affected shoulder and the entire upper limb were prepared and draped in the usual sterile fashion. Prior skin incisions were delineated with a sterile surgical marking pen. In the case of a previous osteosynthesis, laboratory evaluation with erythrocyte sedimentation rate (ESR) and C-reactive protein (CRP) had always taken place. Furthermore, intraoperative tissue samples for culture were obtained and perioperative antibiotics including cefuroxime and vancomycin were given IV for at least 48 hours.

In the case of nonunion, the ipsilateral iliac crest or fibula were also draped for graft harvesting. Corticocancellous graft from the anterior iliac crest was harvested and subsequently introduced to the nonunion site after meticulous preparation of bone surfaces. When a segmental defect and extended bone loss were apparent, a free fibula strut graft of appropriate length was applied to enhance the stability of plate fixation.

A deltopectoral approach extended distally into Henry's anterolateral approach was utilized in all cases [15]. The long head of the biceps was identified, and the periosteum was incised longitudinally, staying lateral to the tendon. Furthermore, part of the insertion of the pectoralis major tendon (less than 1 cm) was detached subperiosteally and laterally to facilitate proper bone exposure for osteosynthesis. The anterior one-third of the deltoid insertion was also elevated subperiosteally to allow positioning of the plate lateral to the bicipital groove [11,15]. At the humeral diaphysis, the brachialis was split longitudinally along its fibers separating its lateral third (radial nerve) from the medial two-thirds (musculocutaneous nerve) of the muscle belly. The radial nerve was routinely explored and identified as blunt insertion of the plate and screws may lead to accidental nerve damage and neurapraxia [5].

**Figure 1:**
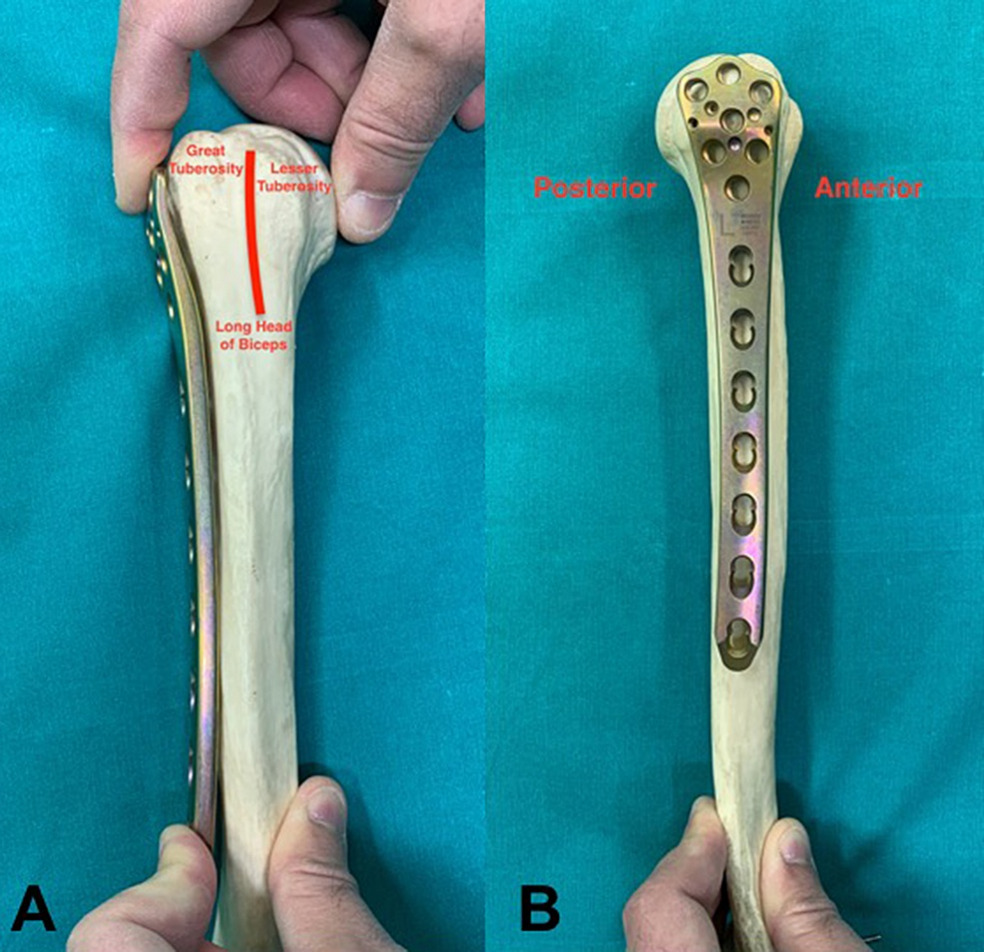
(A) Left 4.5 mm anatomic locking LCP Distal Femur Plate inserted to the right humerus with the splayed part of the plate facing proximally and posteriorly. (B) The correct plate position is at least 5 to 8 mm distal to the top of the greater tuberosity and slightly posterior to the bicipital groove to avoid anterior impingement and irritation of the tendon of the long head of the biceps.

The fracture was reduced with manipulation movements and traction to achieve provisional alignment and stability. Depending on fracture configuration and size of bone fragments, 4.5 mm lag screws were inserted. The next step included the introduction of the plate on the lateral surface of the humerus. The type of implant used was the contralateral 4.5 mm anatomic locking LCP Distal Femur Plate (DePuy Synthes, West Chester, USA) with the splayed part of the plate facing proximally (Figure 1). 

The concept of using the contralateral distal femur plate (left to right and vice versa) was based on plate configuration as its offset should face posteriorly to avoid anterior impingement and irritation of the long head of the biceps tendon. The “racket” part of the plate allows the introduction of a large number of screws in order to optimize proximal fixation. The correct plate position was at least 5 to 8 mm below the proximal tip of the greater tuberosity and slightly posteriorly to the bicipital groove (2 to 4 mm). The plate was aligned to the humeral shaft and its length was selected so that at least 3 screw holes were available at the distal fracture fragment [11]. In case of significant fracture comminution, a plate reduction technique was applied ensuring correct anterior-posterior alignment of the implant. The plate was affixed to the distal bone fragment by using a non-locking screw and then the metaphysis and proximal humerus were reduced onto the plate. At this point, an image intensifier was used to confirm fracture reduction and accurate plate position.

At least four locking screws were introduced proximally at the humeral head and metaphyseal region. Special attention was given to achieve calcar restoration for maintaining fracture reduction and avoiding fixation failure [2]. Distal to the fracture plane, the remaining screws were inserted in a locking bicortical fashion to optimize construct strength. Drill sleeves were used throughout the whole procedure to avoid the possibility of winding the radial nerve up in the drill. Similarly, Hohmann-type retractors were avoided at the midshaft and distal diaphysis region to reduce the risk of nerve injury. If the fracture lines extended into the tuberosities, Ethibond No 2 sutures (Ethicon, Somerville, NJ, USA) were placed in the insertion of supraspinatus/ infraspinatus and subscapularis tendons on the great and lesser tuberosities, respectively. After fracture reduction and proper plate position, the sutures were passed through the proximal holes of the plate and subsequently tied to secure the tuberosities to the shaft. At the end of the procedure, multiple fluoroscopic views were used to confirm the length of the screws and avoid any penetration of the articular surface of the humeral head.

The deltopectoral interval and the space between biceps and mobile wad were reapproximated with interrupted 1 Vicryl absorbable sutures (Ethicon, Somerville, NJ, USA). Subcutaneous tissues were closed with 2.0 Vicryl sutures and staples were applied for skin closure.

Postoperatively, a plaster U-shaped splint with padding under the axilla was introduced and wrapped from medial to lateral and over the shoulder. After two weeks, the splint was removed, and elbow active and passive motion exercises were commenced. Passive shoulder range of motion exercises were encouraged nearly one month postoperatively. Active shoulder exercises along with strengthening of the deltoid and rotator cuff muscles were suggested after the first signs of fracture healing at approximately 2 months after surgery. All patients were examined and evaluated for fracture healing and functional outcome using the DASH score process every month for at least 12 months [16].

## Results

There were eight women and three men. All patients were over 50 years old and with a mean age of 70 years (range, 52 to 84 years). The dominant hand was involved in seven out of eleven patients. The mean follow-up was 13 months (range, 12 to 15 months).

**Figure 2:**
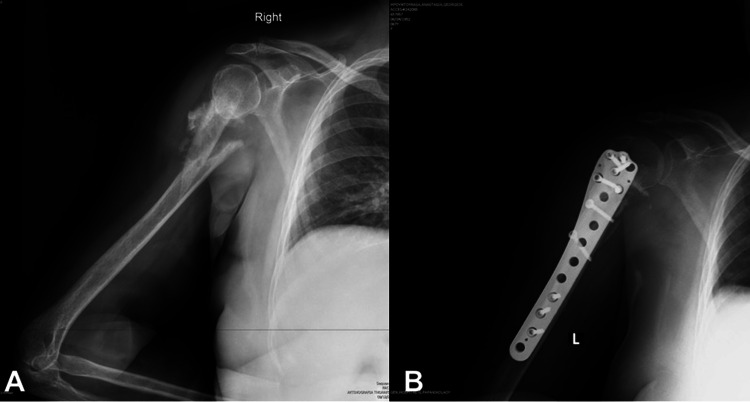
(A) Nonunion of a proximal metadiaphyseal humerus fracture. (B) Reversed anatomic distal femoral locking plate technique for fracture fixation.

There were seven acute metadiaphyseal fractures (Figure 2) and four nonunions.

**Figure 3:**
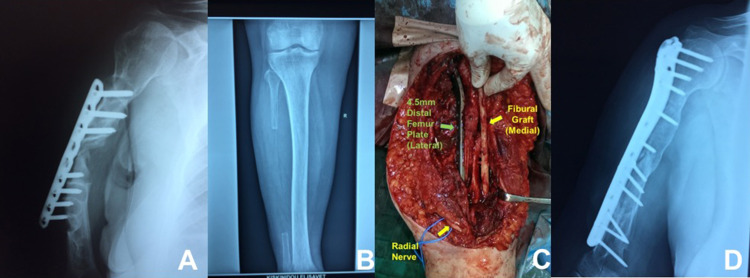
(A) Nonunion and failed internal fixation of a humeral shaft fracture. Poor bone stock with a segmental defect is evident until the proximal metaphysis. (B) Harvesting of a free non-vascularized fibular graft from the ipsilateral limb. (C) Internal fixation with a long reverse distal femoral plate and augmentation of fracture site with the longitudinally split fibular graft. (D) Four months postoperatively the fracture has healed.

In two out of four nonunion cases a previous open reduction internal fixation (ORIF) was applied. The mean time interval between initial fracture treatment (conservative or surgical) and nonunion operation was 7.3 months (range, 6 to 9 months). In one patient, humeral shaft nonunion was associated with segmental metadiaphyseal defect and a free fibular graft was applied (Figure 3).

The surgical procedures were successfully completed in all cases without intraoperative complications. The mean operative time was 132 minutes (95 to 181 minutes). Intraoperative blood loss was 111.7 mL (range, 90 to 152 mL). No major trauma or systemic complications were recorded. No local complications were noted during the short-term follow-up. 

Both acute fractures and nonunions healed clinically and radiographically. The mean time of fracture healing was 10±1.5 weeks for acute fractures and 12.2±2.9 for nonunions.

All patients regained almost normal function of the affected shoulder and upper limb. Specifically, shoulder abduction and forward elevation ranged from 80 to 110 degrees (mean, 97 degrees) and 90 to 120 degrees (mean, 102 degrees), respectively. The final DASH score varied from 6 to 11 points (median 8).

## Discussion

Both nailing and plating techniques have been described so far for the treatment of displaced proximal metadiaphyseal humerus fractures with reasonable results [1-3,6,17]. Although minimally invasive approaches can be applied using indirect methods for fracture reduction, a single extensile approach is usually necessary [1]. However, and due to the rarity of this fracture pattern only case reports and case series including a small number of patients existed in the literature. Therefore, the effectiveness of each technique for achieving bone union could not be clearly defined as nonunion rates of up to 12% have been reported [3]. Our fixation method has been associated with a complete union of both acute fractures and nonunions and could be considered an alternative option for the surgical treatment of this uncommon type of injury.

The primary indications of the presented technique are fractures of the proximal humerus that extended distally at the humeral shaft. Particularly, comminuted and segmental fractures that have a higher possibility of nonunion are more suitable for utilization of the current surgical procedure. It can be also selected in cases of nonunions and previously failed fixations with intramedullary nails or 3.5 mm PHILOS-type plates. In this scenario, additional iliac crest autograft with or without augmentation with strut grafts should be applied to facilitate fracture healing [18]. Apart from metadiaphyseal fractures, humeral shaft fractures and nonunions with poor bone stock that require long plate fixation and multiple screws insertion to proximal metaphysis and humeral head for achieving construct stability are also candidates for application of the method [17].

The major contraindication of the technique is infective nonunion and intraarticular comminuted fractures of the humeral head not amenable to open reduction and internal fixation. In the first case, previous implant removal should be followed by a 6 to 12 weeks period of antibiotics and subsequent new fixation with the described technique. Comminuted displaced intraarticular fractures with metaphyseal extension in the elderly, that are not amenable to internal fixation, can be better treated with hemiarthroplasty or reverse total shoulder arthroplasty using long cemented stems and supplementary cerclage wiring of displaced metaphyseal bone fragments [19].

Several potential complications of the method can be mentioned. Radial nerve palsy may be encountered particularly after vigorous manipulation of the distal humerus and forceful retraction such as with a Hohmann retractor [14,20]. Less often, musculocutaneous nerve injury during retraction of biceps and postoperative neuroma of the lateral cutaneous nerve may occur. Infection and wound problems are also a possible scenario particularly due to the extensile character of the approach and preceding surgical interventions. Metalware irritation could be further hypothesized as the 4.5 mm anatomic distal femoral plate is stiffer and bulkier than the 3.5 mm proximal anatomic shoulder plates. However, the adequate coverage of the plate from the deltoid, biceps, and mobile wad muscle bellies minimizes the potential of hardware prominence and soft tissue irritation. So far, neither tenderness nor metalware removal due to prominence has been encountered even 3 years after surgery.

Study limitations

The present study has certain limitations. It represents a small pilot case series study with a relatively small sample size. Moreover, a direct comparison of the technique to the conventional methods was not performed. Another weakness of the study is the heterogeneity of the patients, as both patients with acute fractures and nonunions were included. However, reporting the results for both makes a point that the described technique can be utilized for all cases of proximal humeral fractures and nonunions with metaphyseal and diaphyseal extension

## Conclusions

Operative treatment of displaced metadiaphyseal fractures with or without extension to the humeral head is a challenging and difficult procedure. The 3.5 mm anatomic plates don’t offer the same strength and stability compared to 4.5 mm plates especially in the humeral shaft region. Therefore, the application of a 4.5 mm plate that fits properly to the anatomy of proximal humerus would increase the potential of healing of both acute and nonunited fractures. The described technique is more useful in case of osteoporosis and/or the presence of previous failed internal fixation that further compromises the vascularization and the mechanical properties of the bone.
